# Synaptic inputs to displaced intrinsically-photosensitive ganglion cells in macaque retina

**DOI:** 10.1038/s41598-022-19324-z

**Published:** 2022-09-07

**Authors:** Andrea S. Bordt, Sara S. Patterson, James A. Kuchenbecker, Marcus A. Mazzaferri, Joel N. Yearick, Emma R. Yang, Judith Mosinger Ogilvie, Jay Neitz, David W. Marshak

**Affiliations:** 1 Department of Neurobiology and Anatomy, McGovern Medical School, Houston, TX USA; 2 Department of Ophthalmology, University of Washington, Seattle, WA USA; 3 Center for Visual Science, University of Rochester, Rochester, NY USA; 4 Department of BioSciences, Rice University, Houston, TX USA; 5 Department of Biology, Saint Louis University, Saint Louis, MO USA

**Keywords:** Neural circuits, Visual system

## Abstract

Ganglion cells are the projection neurons of the retina. Intrinsically photosensitive retinal ganglion cells (ipRGCs) express the photopigment melanopsin and also receive input from rods and cones via bipolar cells and amacrine cells. In primates, multiple types of ipRGCs have been identified. The ipRGCs with somas in the ganglion cell layer have been studied extensively, but less is known about those with somas in the inner nuclear layer, the “displaced” cells. To investigate their synaptic inputs, three sets of horizontal, ultrathin sections through central macaque retina were collected using serial block-face scanning electron microscopy. One displaced ipRGC received nearly all of its excitatory inputs from ON bipolar cells and would therefore be expected to have ON responses to light. In each of the three volumes, there was also at least one cell that had a large soma in the inner nuclear layer, varicose axons and dendrites with a large diameter that formed large, extremely sparse arbor in the outermost stratum of the inner plexiform layer. They were identified as the displaced M1 type of ipRGCs based on this morphology and on the high density of granules in their somas. They received extensive input from amacrine cells, including the dopaminergic type. The vast majority of their excitatory inputs were from OFF bipolar cells, including two subtypes with extensive input from the primary rod pathway. They would be expected to have OFF responses to light stimuli below the threshold for melanopsin or soon after the offset of a light stimulus.

## Introduction

In primates, there are multiple types of retinal ganglion cells that differ in their morphology, synaptic inputs, light responses and central projections^1^. These include intrinsically photosensitive retinal ganglion cells (ipRGCs), which express the photopigment melanopsin and respond to light directly. They also receive input from rods and cones via excitatory synapses from bipolar cells and synapses from amacrine cells that are typically inhibitory. Their central projections include the suprachiasmatic nucleus of the hypothalamus, the dorsal lateral geniculate nucleus and the pretectal olivary nucleus^2^.

Mice have at least six types of ipRGCs with distinct morphology, light responses and central projections^3^. However, it is uncertain how many types of ipRGCs exist in primates. Initially, the primate ipRGCs were divided into two types: outer-stratifying cells, also known as M1, whose dendrites branched in the first stratum (S1) of the inner plexiform layer (IPL), and inner-stratifying cells, also known as M2, whose dendrites branched in the fifth stratum (S5)^4^. More recent studies indicate that there is greater diversity of primate ipRGCs. Using light microscopic immunolabeling in human retina, six types were identified using soma size and position, dendritic arbor size and stratification and the levels of melanopsin expression^5^. Based on patterns of gene expression, three types were identified in peripheral macaque retina^6^. Three types of extracellularly recorded light responses have been observed in human ipRGCs^7^.

We have adopted a different approach to study the diversity of ipRGCs in primates. We reconstructed retinal ganglion cells from images obtained by serial block-face scanning electron microscopy (SBFSEM) of central macaque retina. We identified ipRGCs by their extremely sparse dendritic arbors, their narrow stratification in the IPL and their axonal morphology and then reconstructed the presynaptic neurons. Previously, we identified two types of ipRGCs with somas in the ganglion cell layer (GCL). The first received extensive inhibitory input from short wavelength-sensitive (S) cones via S-ON bipolar cells and S-cone amacrine cells, as well as excitatory input from both ON and OFF bipolar cells. It resembled the M1 morphological type, and its dendrites ramified mainly in S1 of the IPL^8^. A second type resembled the M2 ipRGCs morphologically, with sparse dendrites ramifying entirely in S5 of the IPL. They received direct, excitatory input from ON bipolar cells, almost exclusively from the S-ON type^9^.

Here we report the synaptic inputs to ipRGCs with somas located in the inner nuclear layer (INL), which are also known as displaced ganglion cells. These ganglion cells received the majority of their synaptic inputs from amacrine cells. However, the focus of this study was on their bipolar cell inputs, information that is essential to predict the polarity of their light responses. In each of three volumes taken from different quadrants of the central retina, we found large ipRGCs that ramified narrowly in S1 of the IPL and received excitatory input almost entirely from OFF bipolar cells. These resembled the displaced M1 cells^5^, and they may account for the OFF responses to light reported in a subset of human ipRGCs^7^. In the volume inferior to the fovea, we identified another displaced ipRGC with a smaller soma and some dendrites that descended into the inner half of the IPL. This smaller ipRGC received excitatory inputs almost exclusively from ON bipolar cells, as expected from previous light microscopic studies of displaced ipRGCs in primates^4^.

## Methods

All methods were performed in accordance with the relevant guidelines and regulations.

### Electron microscopy

Male macaque (*Macaca nemestrina*) retinal tissue was obtained from the Tissue Distribution Program at the Washington National Primate Center. All procedures were approved by the Institutional Animal Care and Use Committee at the University of Washington. Central retinal tissue was processed for SBFSEM as previously described^10^. Briefly, three 1 × 1 mm square blocks were fixed in glutaraldehyde, stained *en bloc* with osmium ferrocyanide, uranyl acetate and lead aspartate and then embedded in epoxy resin. The selected areas, ranging from 1.25 to 2 mm from the center of the fovea, were particularly well-suited for connectomic analysis because most of the neurons were small, and the high spatial density of retinal ganglion cells facilitated the identification of rare types. The images were acquired using a Zeiss Sigma VPfield emission scanning electron microscope equipped with a 3View system (Gatan, Inc.). See Table 1 for details.

**Table 1:** Three volumes from central macaque retina were analyzed. Details about the location, section thickness and resolution are listed.

Quadrant	Eccentricity	Section thickness	Number of sections	Dimensions	Resolution
Temporal	~ 2 mm	70 nm	937	220 × 200 µm	7.5 nm/pixel
Inferior	~ 1.5 mm	95 nm	1835	240 × 230 µm	7.5 nm/pixel
Nasal	~ 1.25 mm	50 nm	2354	170 × 180 µm	5 nm/pixel

### Connectomic analysis

Three retinal volumes, sectioned in the horizontal plane and acquired at resolutions of 5 nm/pixel or 7.5 nm/pixel, were studied. Two of these volumes were also used in recent studies of synaptic inputs to other types of retinal ganglion cells^8–12^. Image registration was performed using Nornir (http://nornir.github.io RRID:SCR_003584), and the image tiles were reassembled into cohesive digital volumes and hosted on a 24-core server at the University of Washington.

The serial EM volumes were annotated using the web-based, multiuser Viking software described previously^13^ (RRID:SCR_005986). Briefly, profiles of processes were typically annotated by placing circular discs with the same diameter at their centers of mass and linking them to annotations on adjacent sections. In some instances, neurons were annotated with closed curves in order to provide more realistic reconstructions. Synaptic densities were annotated with lines and linked to the neurons in which they were located. Neurons and other structures were numbered consecutively.

The major cell types were identified using ultrastructural criteria^14–16^. Axon terminals of bipolar cells were filled with synaptic vesicles and contained synaptic ribbons. We confirmed the identity of the presynaptic bipolar cells by reconstructing the axon terminals and, wherever possible, the somas and primary dendrites. Dendrites of amacrine cells contained fewer synaptic vesicles, and they were typically clustered at synapses. Amacrine cell axons, when present, were narrower in diameter and more varicose than the dendrites, and they typically ran for long distances, making synapses *en passant*. Dendrites of retinal ganglion cells were always postsynaptic, and they had axons arising from the soma or a primary dendrite and had large varicosities filled with mitochondria. In all, four ipRGCs and the neurons providing their inputs were annotated.

### Data analysis

Data analysis and three dimensional rendering were performed using an open-source Matlab (Mathworks, RRID: SCR_001622) program https://github.com/neitzlab/sbfsem-tools RRID: SCR_017350 and Tulip (https://tulip.labri.fr/site/). The image rendering was performed using the RenderApp function^17^. Using the SynapseSphere function, synapses were rendered as unit spheres centered at each synapse annotation’s X, Y and Z coordinates then scaled to optimize visibility. Processes of ipRGCs and axon terminals of bipolar cells were analyzed using the IPLDepth function^12^. The boundary between the INL and the inner plexiform layer (IPL) was designated as 0% and the IPL-GCL boundary as 100% depth. For some figures, RenderApp was used to indicate the stratification depth of axons and dendrites using colors of the visible spectrum, with red being the most sclerad and blue the most vitread. For descriptive purposes, the IPL was divided into 5 strata of 20% each, with 1 beginning at the INL and 5 ending at the GCL. The dendrites of ipRGCs were analyzed using the singleDendriteDiameter function^11^, and the soma diameters were determined using the Measure Line tool in Viking.

Figures were prepared using Adobe Photoshop CS6 and SBFSEM-tools. The code and data used to generate the figures in this study will be made available upon request. Alignment and speed of images in Supplemental Fig. 5 were adjusted using Fiji^18^.

## Results

### Displaced ipRGCs

**Figure 1:**
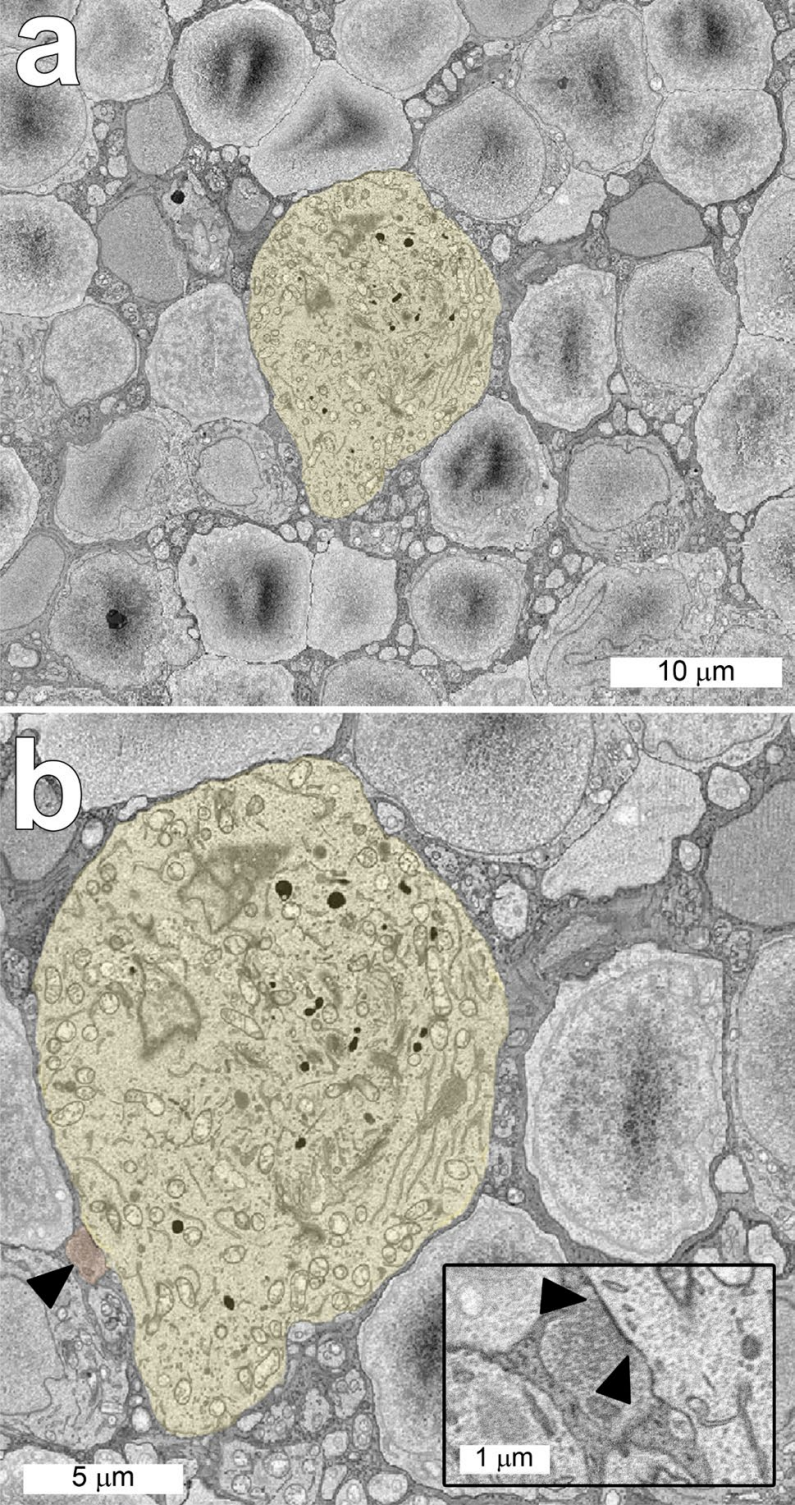
Horizontal sections through the inner nuclear layer of the nasal volume including the soma of displaced ipRGC 11345. (**a**). Note that the diameter of the ipRGC soma (yellow) was much larger than those of the surrounding cells. (**b**). There was a synapse (▲) from an amacrine cell onto the soma of this ipRGC. The boundaries of the synaptic density are indicated at higher magnification in the inset.

The somas of ipRGCs in the INL were clearly distinguishable from those of other types of cells in that layer (Fig. 1). The somas, measured before the primary dendrites had emerged, were considerably larger than those of the surrounding amacrine or bipolar cells, and they contained numerous granules. They also had varicose axons that bifurcated; ipRGCs are the only types of retinal ganglion cells with axons that branch within the retina^19^. Based on these criteria and on their large, extremely sparse dendritic arbors, they were identified as displaced ipRGCs.

**Figure 2:**
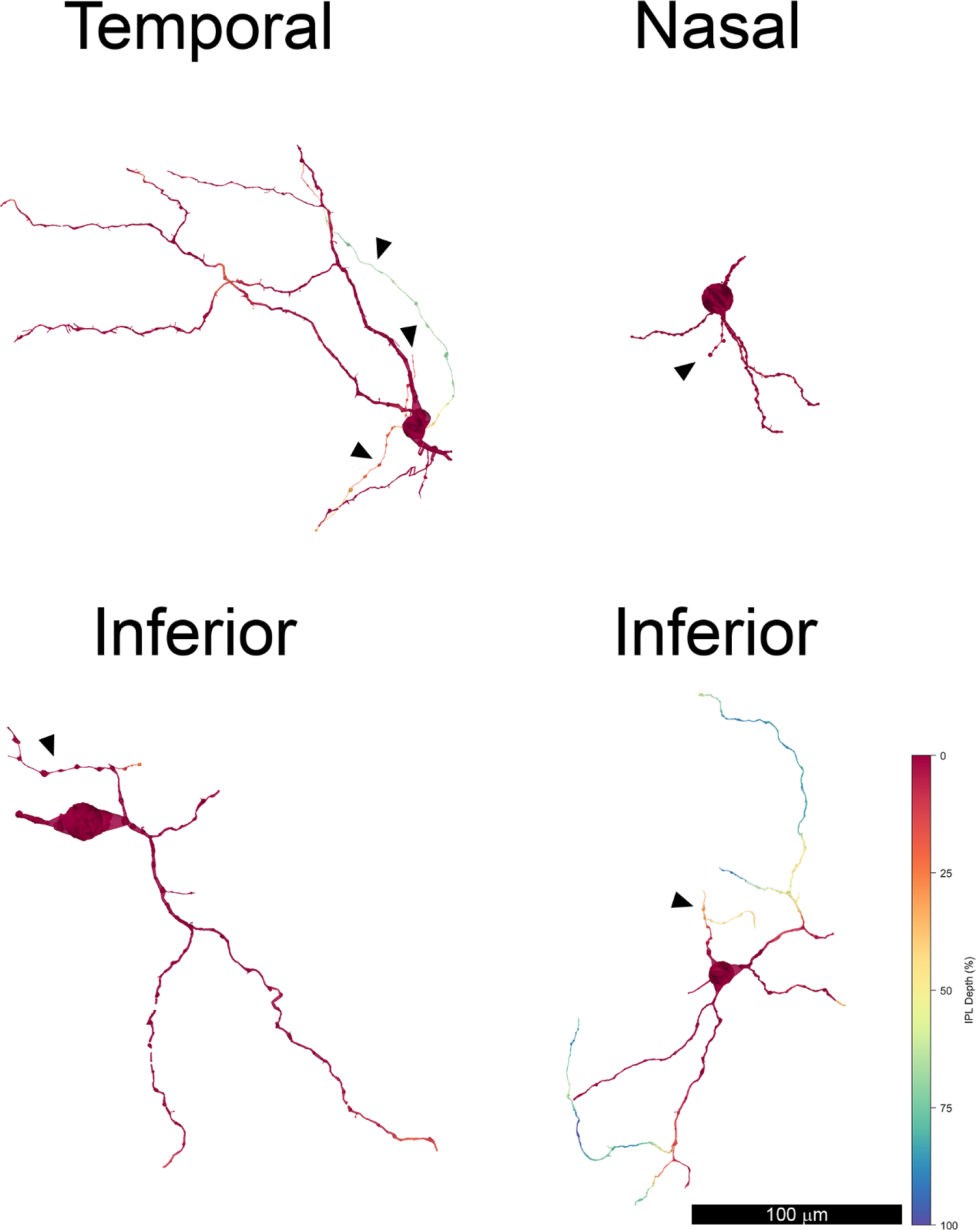
Displaced M1 ipRGCs were reconstructed in each volume: 6210 temporal, 11,345 nasal and 1178 inferior. A smaller displaced ipRGC, 21,551, was also reconstructed in the inferior volume (bottom right). Note that its distal dendrites descended into the inner half of the inner plexiform layer, as indicated by the green and blue pseudocolors. Some dendrites and the soma of cell 6210 from the temporal volume ran off the edges of the volumes, and the axons (▲) were varicose.

The nasal and temporal volumes each contained one displaced ipRGC with a large soma and dendrites confined to S1 of the IPL. In the nasal volume, cell 11345 had an elliptical soma 18.4 by 15.7 µm. In the temporal volume, the soma of cell 6210 was partially truncated, but the intact part measured 14.9 by 12.2 µm (Supplemental Figs. 1.1–1.3). They had varicose axons that arose from the soma or from a primary dendrite and bifurcated (Fig. 2).

The dendrites also had a large diameter, and they branched infrequently. Cell 11345 from the nasal volume had three primary dendrites with mean diameters of 2.58, 2.81 and 1.76 µm; its secondary dendrites had mean diameters of 1.74 and 1.82 µm. Cell 6210 from the temporal volume had primary dendrites with mean diameters of 1.82 and 1.42 µm. The dendritic arbors were large, but it was not possible to determine their exact size because they exceeded the dimensions of the volumes that were analyzed. The dendritic arbors were much larger than those of other types of retinal ganglion cells found at these eccentricities in macaque retinas^4^. These cells were classified as the displaced M1 type^5^.

In the inferior volume, there were two distinct types of displaced ipRGCs, and because they were located nearby, they could be compared directly (Fig. 2). Cell 1178 was illustrated previously^8^, but its synaptic inputs are described here for the first time. It was classified as a displaced M1 type on the basis of its dendritic stratification. The soma was ovoid and its diameter was 19.9 by 15.71 µm. The mean diameter of the short primary dendrite was 2.05 µm. The diameters of the secondary dendrites were 1.21 and 1.36 µm. Cell 21,551 had a smaller soma with a diameter of 15.6 by 13.1 µm. Its primary dendrites were short and had a mean diameter of 1.21 and 1.82 µm. The diameters of its secondary dendrites were: 0.93, 1.18 and 1.07 µm. Its proximal dendrites ramified in S1, and in this respect, it resembled the displaced M1 type^5^. However, three distal dendrites of cell 21551 ramified in S4 and S5. Because there was only a single example, this partially bistratified cell was not classified.

### Presynaptic amacrine cells

**Figure 3:**
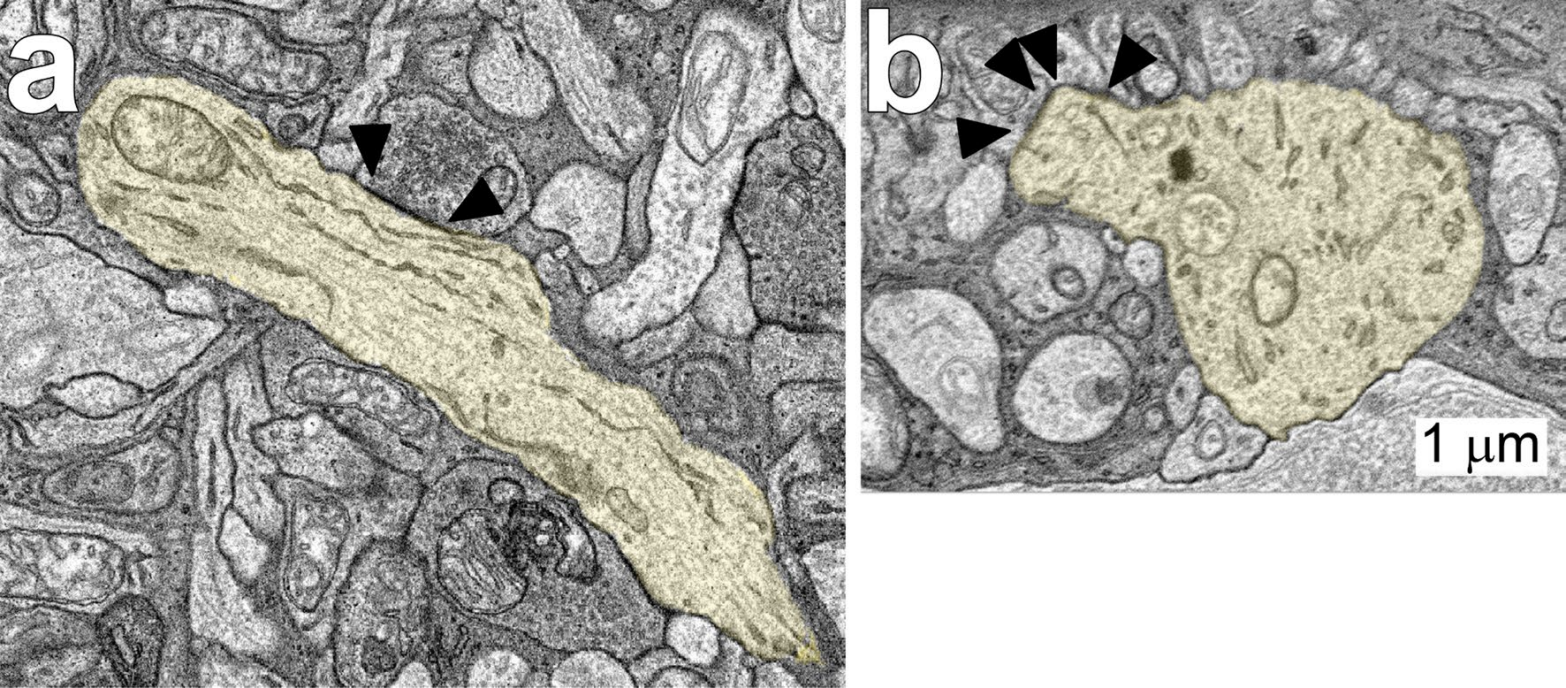
Displaced M1 ipRGC 11,345 (yellow) received synapses (▲) from amacrine cells on its dendrites. Amacrine cells provided the major synaptic input to this and other displaced ipRGCs.

All four displaced ipRGCs received the majority of their synaptic inputs from amacrine cells, and they did not make any synapses within the volumes (Figs. 1, 3). The proportion of input from amacrine cells to the displaced M1 ipRGCs varied in the three quadrants. Amacrine cells were presynaptic at 76% (180/236) of synapses in the nasal volume, 85% in the inferior volume (167/197) and 89% in the temporal volume (330/372). The distributions of amacrine cell synapses onto the four displaced ipRGCs are shown in Supplemental Fig. 3. We reconstructed presynaptic cells amacrine cells sufficiently to determine that the synaptic vesicles were not distributed uniformly and that there were no synaptic ribbons. However, for technical reasons, we were unable to reconstruct the presynaptic amacrine cells sufficiently to classify them.

**Figure 4:**
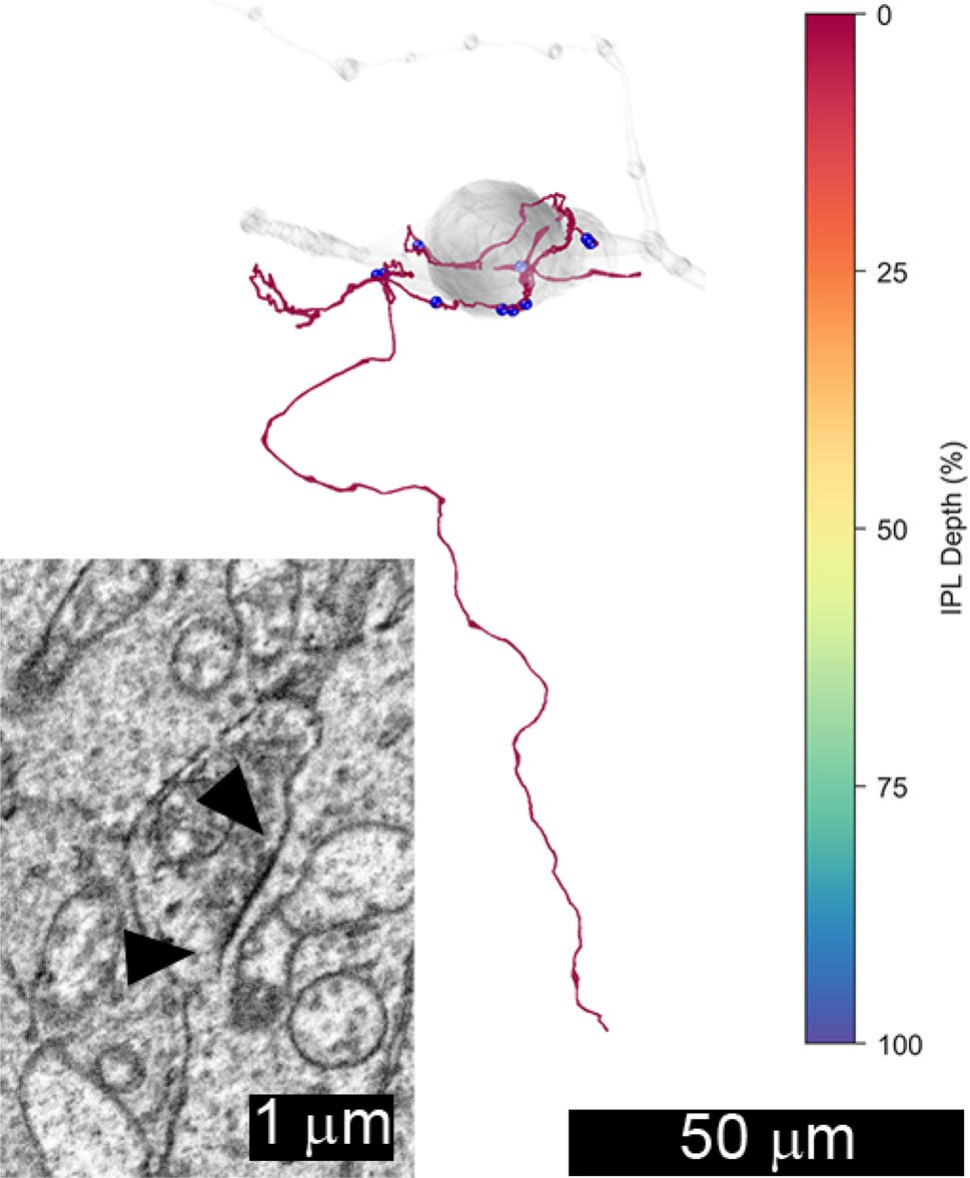
Amacrine cell axon 5560 from the inferior volume was reconstructed. Note its varicose axon and its terminals in the outermost stratum of the inner plexiform layer, S1 (red). It made 12 synapses (●) onto the soma and primary dendrites of ipRGC 1178 (grey). Based on its morphology, it was tentatively identified as an axon of a dopaminergic amacrine cell. The inset shows a typical synapse (▲) from 5560 onto 1178.

Based on previous immunolabeling studies, inputs from dopaminergic amacrine cells were expected^4^. An amacrine cell with the large soma and dendrites ramifying in S1 characteristic of dopaminergic amacrine cells was partially reconstructed in the nasal volume (not illustrated), but no synapses from its dendrites onto the displaced M1 ipRGC were found. One amacrine cell axon that made 12 synapses onto the soma and primary dendrites of ipRGC 1178 in the inferior volume was partially reconstructed (Fig. 4). Based on its pattern of synaptic connections and its narrowly stratified arbor in S1 and the INL, it likely originated from a dopaminergic amacrine cell. Taken together, these findings suggest that dopaminergic inputs to ipRGCs are mediated by the axons originating from somas outside the volume.

### Presynaptic bipolar cells

**Figure 5:**
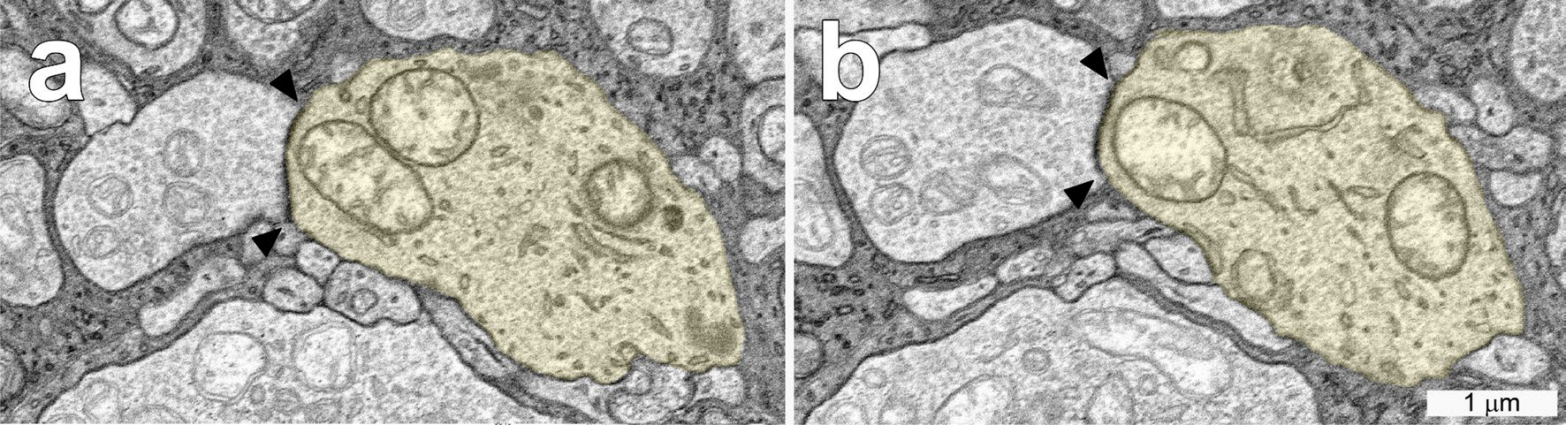
Displaced M1 ipRGC 11345 in the nasal volume (yellow) received input from a bipolar cell axon (11782) at a large non-ribbon synapse (▲). The sections were selected from a series of 18; panel (**a**) Z = 1885, panel (**b**) Z = 1877.

The bipolar cell synapses onto the four displaced ipRGCs are shown in Supplemental Fig. 6. Ribbons surrounded by a halo of vesicles were found at most of the synapses from bipolar cells onto ipRGCs, but they were absent at some of the synapses. This is not attributable to the low contrast of synaptic ribbons in this material. In each instance, the bipolar cells were annotated sufficiently to verify that ribbons were present at other synapses, and many of the presynaptic bipolar cells were identified morphologically. The best-characterized example was found in the nasal volume (Fig. 5). Synaptic densities and vesicles were observed in 18 consecutive 50 nm sections, but there was no associated ribbon. Synaptic ribbons were found elsewhere in the axon terminal of this bipolar cell. In the nasal volume, 66% (41/62) of the bipolar cell synapses onto ipRGC 11354 had synaptic ribbons associated with the synaptic density, but the remainder did not.

**Figure 6:**
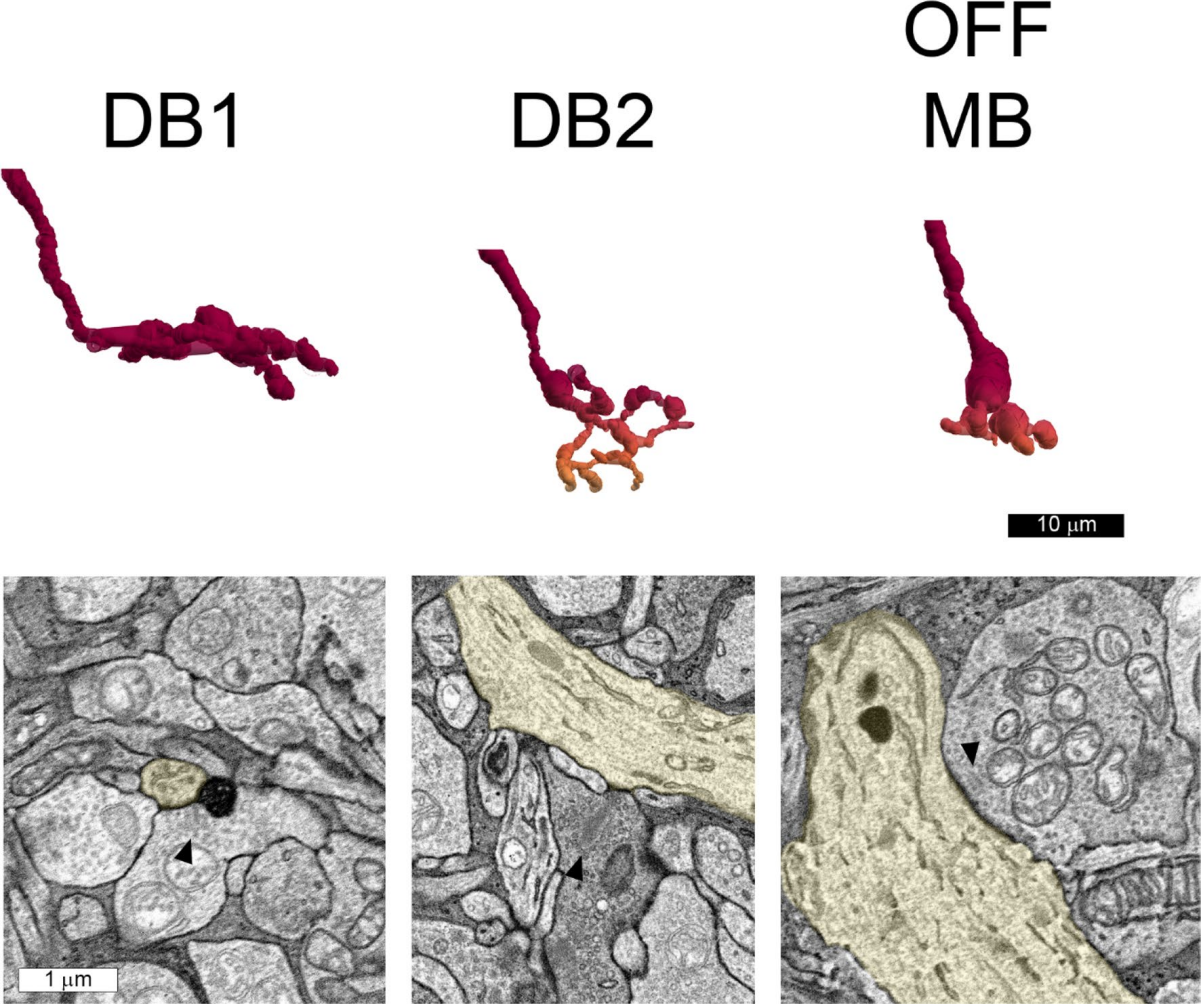
Three types of OFF bipolar cells made synapses onto displaced M1 ipRGC 11345 in the nasal volume. These include diffuse bipolar (DB) cell 1 (11369), DB2 (14615) and OFF midget bipolar (13313) cells. Their reconstructed and pseudocolored axon terminals are shown in the upper panel. Red indicates S1 and orange indicates S2. Note that none of the axon terminals extended into the inner half of the inner plexiform layer. The bipolar cell synapses onto ipRGC 11345 (yellow) are shown in the lower panels, and the synaptic ribbons are indicated (▲).

**Figure 7:**
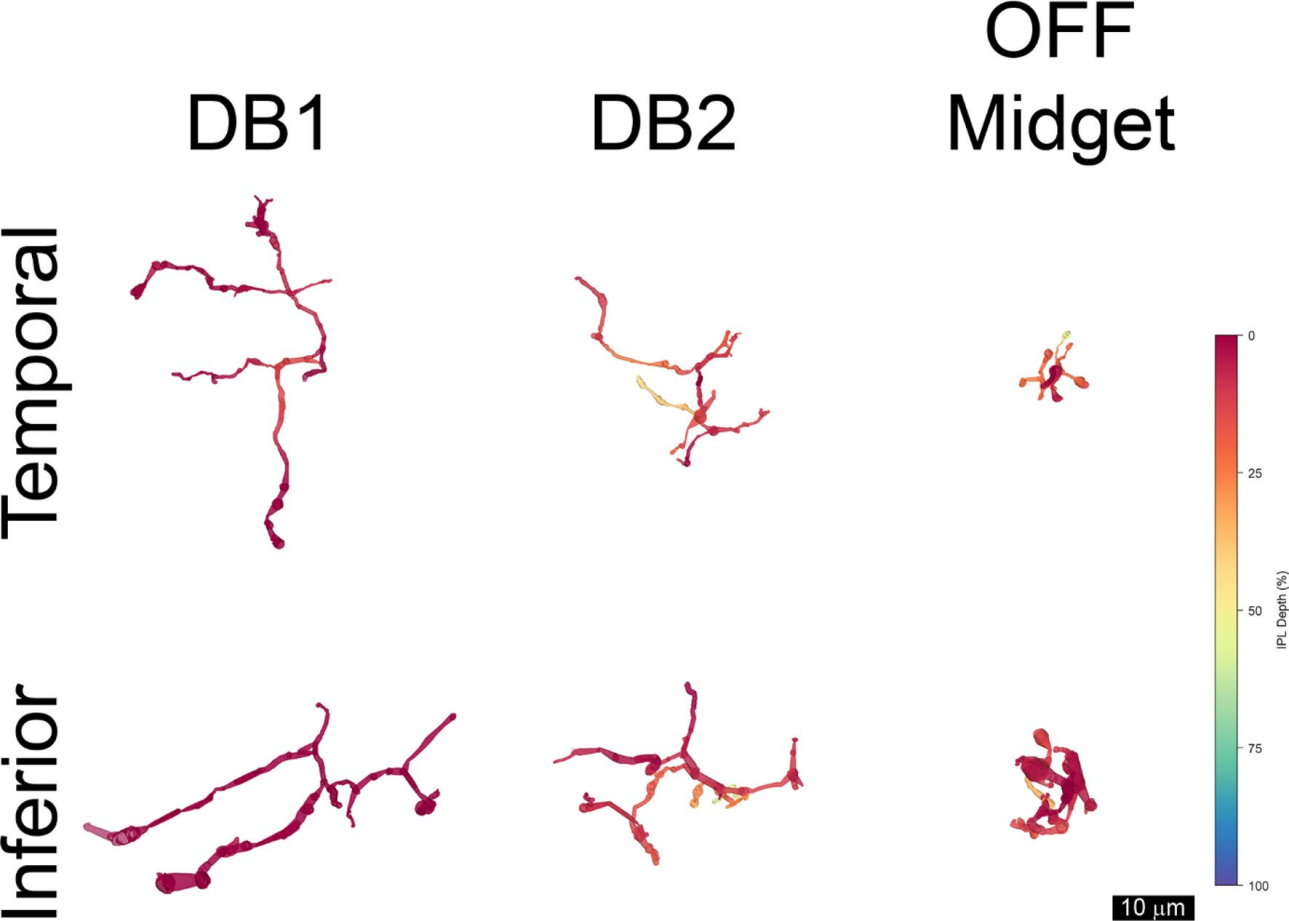
OFF bipolar cell synapses onto displaced M1 ganglion cells in the temporal (t) and inferior (i) volumes. Note that DB1 axon terminals ramified in S1 (red) and that DB2 and OFF midget axon terminals also ramified in S2 (orange and yellow). OFF midget = 44052t, DB2 = 44029t, DB1 = 44289t. DB1 = 48234i, DB2 = 48197i, OFF midget = 945i.

The vast majority of the excitatory inputs to the displaced M1 cells were from OFF bipolar cells. Excitatory inputs to the cell that was reconstructed most completely, 11345 in the nasal volume, came from one presynaptic ON bipolar cell and 27 OFF bipolar cells. The results with the other two displaced M1 cells were very similar. The presynaptic bipolar cells were partially reconstructed and classified based on their morphology^16,20^. Three distinct types of bipolar cells were presynaptic to the displaced M1 ipRGCs (Figs. 6, 7). Diffuse bipolar 1 (DB1) cells had delicate axon terminals that formed arbors with relatively large diameters that were restricted to S1 of the IPL. DB2 bipolar cells had more rugose axon terminals that formed arbors with somewhat smaller diameters that occupied part of S1 as well as S2 of the IPL. Partially-reconstructed DB2 axon terminals could also be identified by their high cytoplasmic electron density (Fig. 6, middle panel). The axon terminals of OFF midget bipolar cells were found in the same strata as those of DB2, but they could be distinguished by the smaller diameters of their axonal arbors and their electron-lucent cytoplasm. Some of the presynaptic bipolar cells could not be identified, but they could be reconstructed sufficiently to determine that they ramified in either S1, S2 or both strata of the IPL.

**Figure 8:**
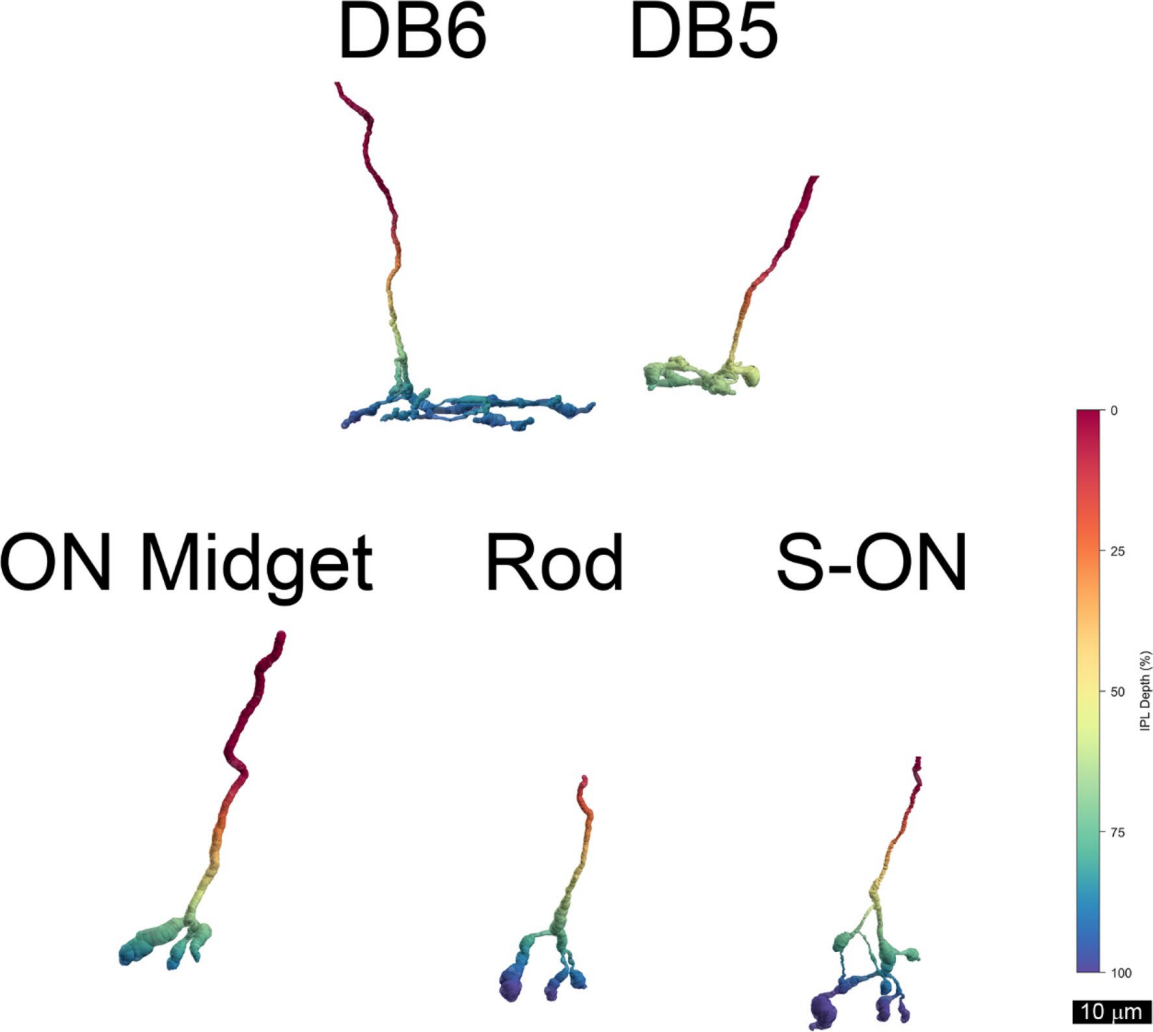
Displaced M1 ganglion cell 2155 received all but one of its excitatory synapses from five types of ON bipolar cells. Note that their axon terminals ramified in S4 (green) and S5 (blue) of the IPL.DB6 = 43031i, DB5 = 52773i, ON midget = 52770i, Rod = 17585i, S-ON = 22103i.

At least four types of bipolar cells were presynaptic to the displaced M1 ipRGC in the inferior connectome, cell 21551 (Fig. 8). One presynaptic bipolar cell was identified as DB1 using the criteria described above, and it made the only synapse from an OFF bipolar cell. The rest of the synapses originated from ON bipolar cells. Two were identified as the S-ON type based on their contacts with small bistratified ganglion cells and S-cone amacrine cells^8,10^. Two other presynaptic bipolar cells that had axonal arbors in S5 with a larger diameter were identified as the DB6 type. One other presynaptic bipolar cell had a large dendritic arbor centered around the border between S4 and S5. It was positively identified as DB5 by reconstructing a population of neighboring DB4 and DB5 bipolar cell axon terminals. Its axon terminal filled a gap in the mosaic of DB5 terminals, but it overlapped with the terminal of a DB4 cell. Four presynaptic bipolar cells with relatively small axon terminals that ramified around the same level in the IPL were classified as ON midget cells^16,20^. One synapse onto the displaced M1 ipRGC was made by a bipolar cell with a very narrow axonal arbor ramifying between 70 and 90% depth in the IPL. It closely resembled the rod bipolar cells identified at the same eccentricity in an electron microscopic immunolabeling study of macaque retina^21^.

## Discussion

We are confident that the ganglion cells reconstructed in this study are ipRGCs based on their distinctive morphology. Moreover, displaced ipRGCs are the only retinal ganglion cells known to be consistently located in the inner nuclear layer; other types of displaced ganglion cells are very rare and likely anomalous^22^. The spatial density of displaced ganglion cells following tracer injection in the optic nerves of macaques is very low^23^, and this is also true of displaced ipRGCs in macaques and humans^4,5,24^.

The majority of the synaptic inputs to the displaced ipRGCs came from amacrine cells. These local circuit neurons are typically inhibitory, but some types may also have excitatory effects^25^. These likely include axon terminals of dopaminergic amacrine cells. In rodents, dopamine directly inhibits ipRGCs via D1 receptors^26^. DRD1 is the major dopamine receptor gene expressed by macaque ipRGCs^6^, and we predict that dopamine would have a similar effect. However, the focus of this study was on the input to ipRGCs from bipolar cells.

The displaced M1 ganglion cells of human retina are a distinct type based on their morphology^5,22^, and the data reported here on the preponderance of excitatory synaptic inputs from OFF bipolar cells to their homologs in macaque retina support this finding. This is the first study to identify the types of bipolar cells that provide input to displaced ipRGCs using electron microscopy (EM). Some of these synapses from bipolar cells were not associated with synaptic ribbons (Fig. 5, Table 2). In this respect, they were similar to synapses described previously using EM. In salamander and rabbit retinas, synapses like these are seen using serial, ultrathin sections^27–29^. These synapses may also account for the images of excitatory synapses without synaptic ribbons observed in macaque retinas using freeze-fracture^30^.

**Table 2:** Bipolar cells presynaptic to displaced ipRGC 11345 from the nasal volume. Note that input from type DB1 predominated. Each type of presynaptic bipolar cell made at least one synapse without a ribbon. The presynaptic bipolar cells could not be identified at 12 synapses. Together, these bipolar cells made 62 synapses onto ipRGC11345. Of these 66% (41/62) had ribbons and 34% (21/62) did not.

Cell type	OFF midget	ON midget	DB1	DB2
Number of ribbon synapses	3		23	5
Number of non-ribbon synapses	5	1	8	5

The displaced M1 cells would be expected to generate rapid, transient excitatory responses to decrements in light intensity and, initially, very little response to light increments. If the stimulus intensity exceeded the threshold to activate melanopsin, a sustained, excitatory response would be expected to follow after a delay. Additional experiments using intracellular recordings with tracer injections in macaque retina would be required to confirm the identity of the cells, but, all the published recordings using these techniques in primate retinas have been from ipRGCs with somas in the GCL^31^. Transient OFF responses mediated by inputs from bipolar cells have been described in a subset of human ipRGCs recorded extracellularly with multi-electrode arrays^7^. However, because M1 cells with somas in the GCL also receive some excitatory inputs from OFF bipolar cells^8^, it is uncertain whether those responses were generated by neurons with somas in the GCL or in the INL. If they project to the same targets in the brain, the OFF responses from ipRGCs would be expected to oppose the actions of ON responses of the other ipRGCs.

The excitatory inputs to displaced M1 ipRGCs came from three different types of OFF bipolar cells: DB1, DB2 and OFF midget. These would be expected to respond to decreases in light intensity under a wide range of ambient light intensities. In scotopic conditions, they would receive signals from the primary rod pathway via AII amacrine cells, which provide extensive input to DB1 and OFF midget bipolar cells. Under photopic conditions, those bipolar cells would receive crossover inhibition from the AII cells and convey this to the ganglion cells^32^. Both DB1 and OFF midget bipolar cells have sustained responses to light mediated by their voltage-gated conductances and the kainate receptors on their dendrites. Different voltage-gated conductances and kainate receptors of DB2 cells generate rapid, transient responses^32–34^. Together, the three types of OFF bipolar cells would generate robust responses to decrements in light intensity in displaced M1 ipRGCs.

The displaced M1 ganglion cell 21551 in the inferior volume received the majority of its excitatory input from multiple types of ON-type cone bipolar cells, including: S-ON, ON midget, DB5 and DB6 bipolar cells. These inputs would be expected to generate light responses in the displaced M1 ganglion cell under a wide variety of conditions. The ON midget bipolar cells receive input from the primary rod pathway via AII amacrine cells^35^. The DB5 bipolar cells have T-type voltage-gated calcium currents and are expected to have transient responses to light onset. Based on their voltage-gated currents, DB6 and ON midget ganglion cells are expected to have more sustained responses to light onset^33^. S-ON bipolar cells have not been studied directly, but, based on their expression of metabotropic glutamate receptors and the responses of their postsynaptic cells, they are expected to have sustained ON responses to short wavelength lights in their receptive field centers^36,37^.

Previous light microscopic studies found that DB6 bipolar cells contacted ipRGCs^4,38^ and we confirmed that these contacts are synapses. One of the excitatory synapses onto displaced M1 ganglion cell 21551 was made by a rod bipolar cell. The same was true of an M1 ipRGC with its soma in the GCL^8^. This finding had also been predicted using light microscopic immunolabeling in human retinas^5^. However, in other studies of macaque retinas, no contacts between immunolabeled ipRGCs and rod bipolar cells were observed, and rod bipolar cells did not make synapses onto ganglion cell dendrites^21,38^. This discrepancy may be attributable to the low spatial density of displaced M1 ipRGC somas and their sparse dendritic arbors. As a result, synapses like these would be very rarely encountered. Only one of the excitatory inputs in our study was from an identified rod bipolar cell, and in a study of seven fully-reconstructed rod bipolar cells in the rabbit retina, only two synapses from rod bipolar cells onto retinal ganglion cells were reported^28^.

The visual input for the pupillary reflex is mediated by projections of ipRGCs to the olivary pretectal nucleus^39,40^. Melanopsin responses are relatively slow, and, in humans, inputs from rods contribute to the early component of pupillary dilation that follows the offset of light^41^. Light offset would be an optimal stimulus for ipRGCs with OFF responses mediated by the primary rod pathway. We propose that the displaced M1 ipRGCs provide visual input to the pupillary control mechanism that drives dilation of the pupil^42^. The contributions of these ipRGCs to pupillary light responses would be particularly important at light intensities below the threshold of melanopsin. In humans, rod inputs via ipRGCs contribute to steady-state pupillary constriction under these conditions^43^.

## Conclusions

The major finding of this study was that three displaced ipRGCs received virtually all of their excitatory input from OFF bipolar cells, mainly from the two types of bipolar cells known to receive input from the primary rod pathway^32^. No recordings from identified displaced ipRGCs have been published, but our findings suggest that these ipRGCs would initially depolarize and fire action potentials in response to decrements in light intensity. Intracellular recording followed by tracer injection would be required to test this hypothesis.

## Supplementary Information

Supplementary Information 1.

Supplementary Information 2.

## Data Availability

The datasets analyzed during the current study are available to the public on a read-only basis (http://connectomes.utah.edu). The volume addresses are: inferior: http://v2486.host.s.uw.edu/Neitz/InferiorMonkey/SliceToVolume.vikingxml. temporal: http://v2486.host.s.uw.edu/Neitz/TemporalMonkey2/SliceToVolume.vikingxml. nasal: http://v2486.host.s.uw.edu/Neitz/NM/SliceToVolume.vikingxml.
