# Supplementary material for: Synaptic inputs to displaced intrinsically-photosensitive ganglion cells in macaque retina
Source: Sci Rep. 2022 Sep 7;12:15160. doi: 10.1038/s41598-022-19324-z (PMC9452553; doi:10.1038/s41598-022-19324-z)
Supplement: Supplementary file 1 — Supplementary Information 1. [file 41598_2022_19324_MOESM1_ESM.docx]

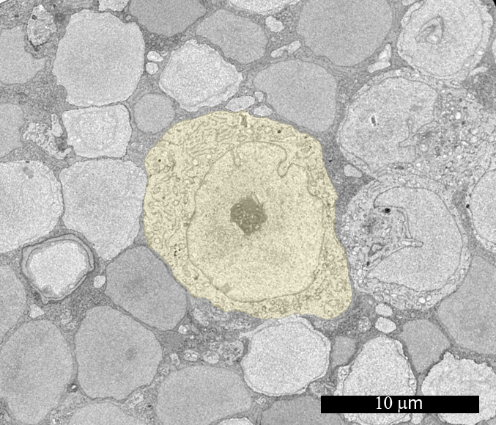


Supplemental Figure 1.1. The soma of cell 6210 (yellow) is surrounded by smaller somas of bipolar cells and amacrine cells in the INL.


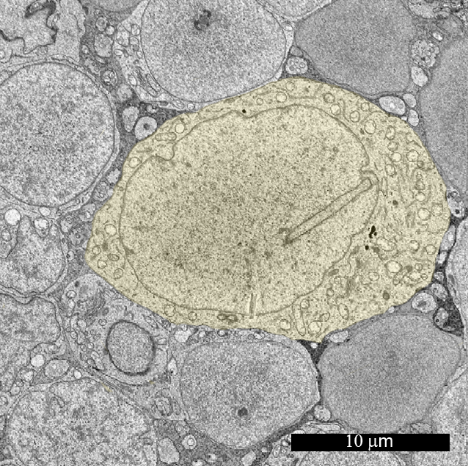


Supplemental Figure 1.2. The soma of cell 1178 (yellow) is surrounded by smaller somas of bipolar cells and amacrine cells in the INL.


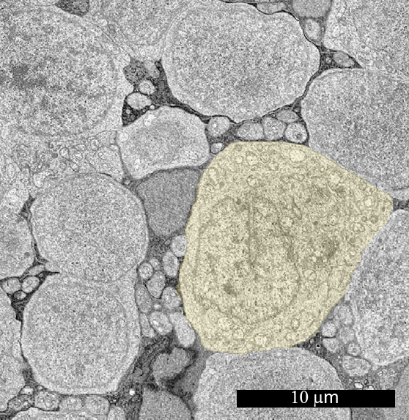


Supplemental Figure 1.3. The soma of cell 21551 (yellow) is surrounded by smaller somas of bipolar cells and amacrine cells in the INL.


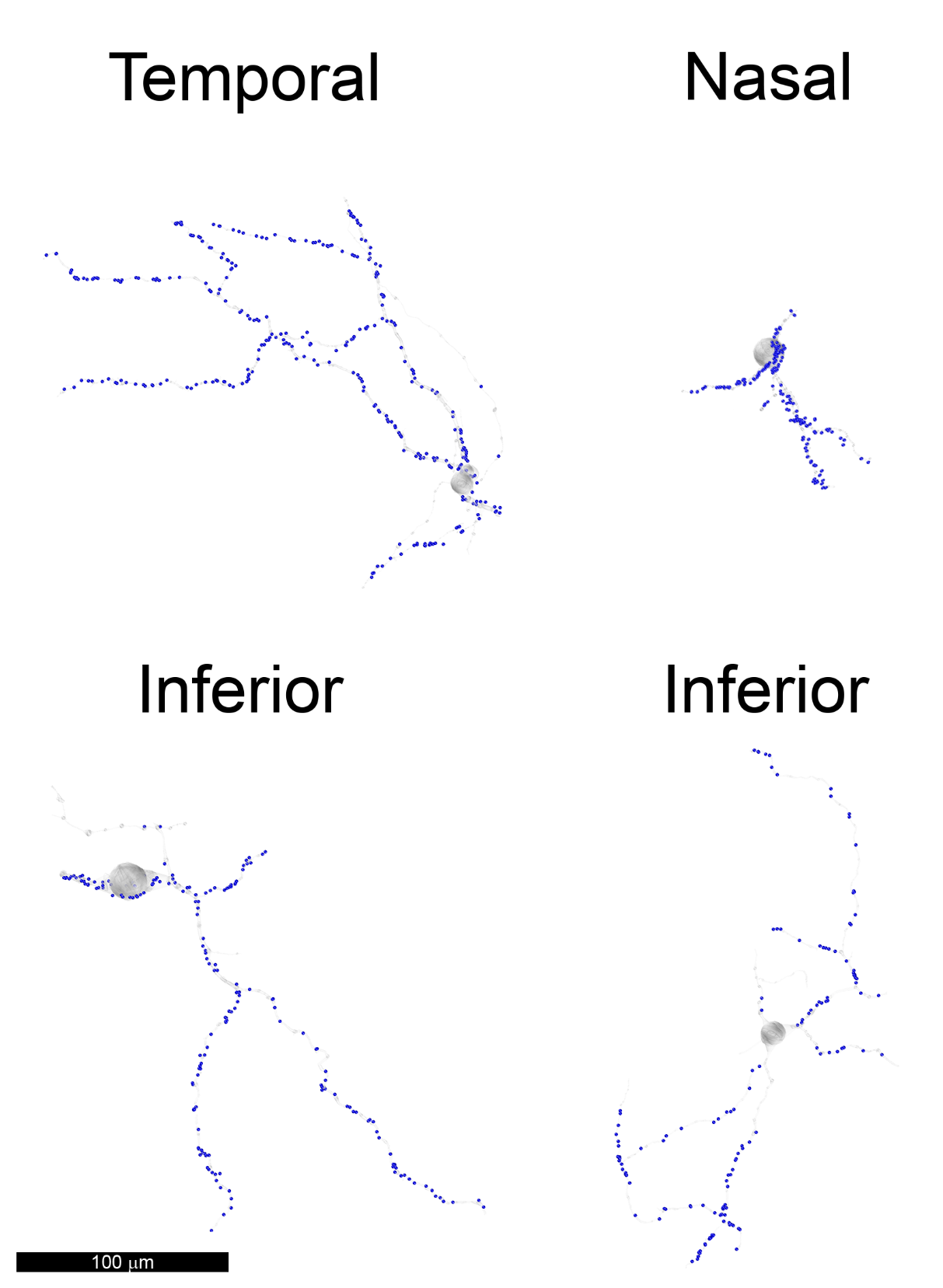


Supplemental Figure 3. Amacrine cell inputs (●) to the four ipRGCs. Displaced M1 ganglion cell 1178i is on the lower left and displaced ganglion cell 21551i is on the lower right. Amacrine cell synapses were uniformly distributed onto dendrites of all four cells. Note that the displaced M1 cells had amacrine cell inputs to their somas, but displaced ipRGC 21551i did not.


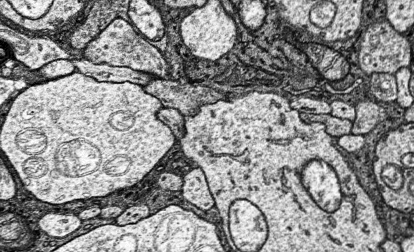


Supplemental Figure 5. GIF movie (30 images) of the non-ribbon synapse shown in Figure 5. Note that there are no synaptic ribbons associated with this synapse. Ribbons were found elsewhere in the bipolar cell.


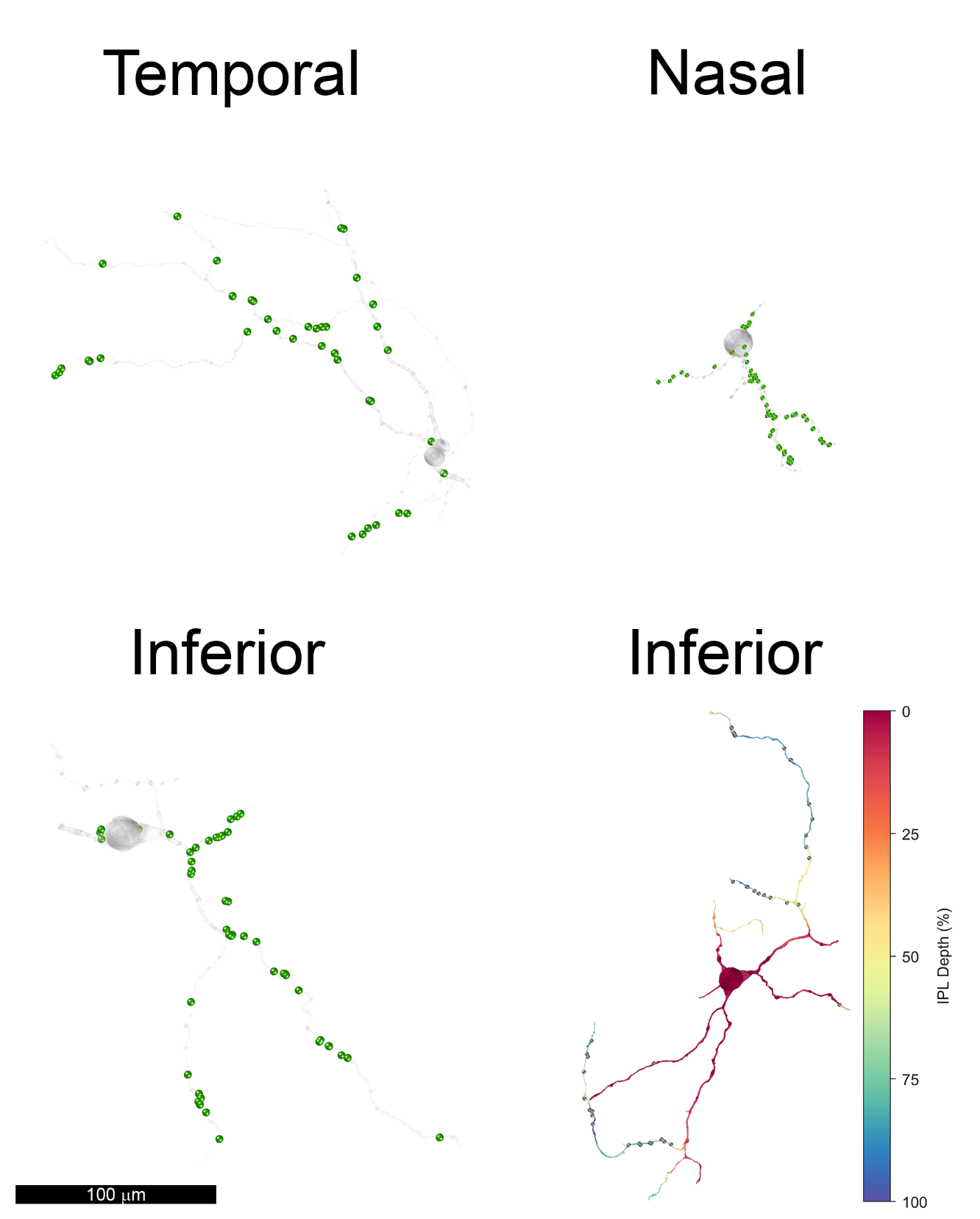


Supplemental Figure 6. Bipolar cell inputs (●) to the soma and dendrites of the four ipRGCs. The displaced M1 cells are grey and the bipolar cell synapses onto them are green. The partially bistratified ipRGC is pseudocolored by depth and the bipolar cell inputs are in black. This cell received bipolar cell input almost exclusively on the dendrites that descended into the inferior half of the IPL.
