# Supplementary figures and images for: Synaptic inputs to displaced intrinsically-photosensitive ganglion cells in macaque retina
Source: Sci Rep. 2022 Sep 7;12:15160. doi: 10.1038/s41598-022-19324-z (PMC9452553; doi:10.1038/s41598-022-19324-z)

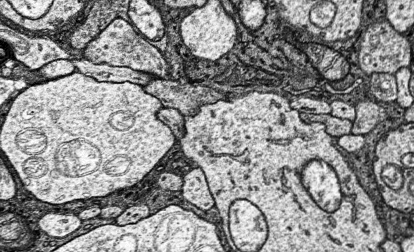

Supplement: Supplementary file 2 — Supplementary Information 2. [file 41598_2022_19324_MOESM2_ESM.gif]
